# Validation and refinement of gene-regulatory pathways on a network of physical interactions

**DOI:** 10.1186/gb-2005-6-7-r62

**Published:** 2005-07-01

**Authors:** Chen-Hsiang Yeang, H Craig Mak, Scott McCuine, Christopher Workman, Tommi Jaakkola, Trey Ideker

**Affiliations:** 1 Center for Biomolecular Science and Engineering, Baskin School of Engineering, University of California at Santa Cruz, Santa Cruz, CA 95064, USA; 2 Department of Bioengineering, University of California at San Diego, La Jolla, CA 92093, USA; 3 Computer Science and Artificial Intelligence Laboratory, Massachusetts Institute of Technology, Cambridge, MA 02139, USA

## Abstract

As genome-scale measurements lead to increasingly complex models of gene regulation, systematic approaches are needed to validate and refine these models. Towards this goal, we describe an automated procedure for prioritizing genetic perturbations in order to discriminate optimally between alternative models of a gene-regulatory network. Using this procedure, we evaluate 38 candidate regulatory networks in yeast and perform four high-priority gene knockout experiments. The refined networks support previously unknown regulatory mechanisms downstream of *SOK2 *and *SWI4*.

## Abstract 2

A new automated procedure for prioritizing genetic perturbations was used to evaluate 38 candidate regulatory networks in yeast. Further analysis of four high-priority gene knockout experiments provided new insights into two regulatory pathways

## Background

Recent advances in genomics and computational biology are enabling construction of large-scale models of gene-regulatory networks. High-throughput technologies such as automated sequencing [1], gene-expression arrays [2], chromatin immunoprecipitation [3], and yeast two-hybrid assays [4], each probe different aspects of the gene-regulatory system through genome-wide datasets. These data have spawned a variety of methods to infer the structure of gene-regulatory networks or to study their high-level properties, as recently reviewed [5].

Regulatory network models generated thus far in *Escherichia coli *and budding yeast (*Saccharomyces cerevisiae*) have been most often validated against functional databases or previous literature [6,7]. In contrast, only a few studies have attempted to validate or refine models systematically [8-11]. However, if we are to accurately model large gene networks in complex organisms, including fly, worm, mouse, and human, automated procedures will be essential for analyzing the network, choosing the best new experiments to test the model, conducting the experiments, and integrating the resulting data.

The problem of choosing the best experiments to estimate a model, termed 'experimental design' or 'active learning', has been a significant area of research in statistics and machine learning [12-14]. Automating the experimental design process can greatly accelerate data collection and model building, leading to substantial savings in time, materials, and human effort. For these reasons, many industries such as electronic circuit fabrication and airplane manufacturing incorporate experimental design as an integral step in the design process [15,16]. A promising application of experimental design for biological systems was presented by King *et al*. [17], who integrated computational modeling and experimental design to reconstruct a small, well studied metabolic pathway. Whether automated experimental design can be useful in a large and poorly characterized biological system with noisy data remains an open question.

We recently reported a procedure for inferring gene-regulatory network models by integrating gene-expression profiles with high-throughput measurements of protein interactions [18]. Here we extend this procedure to incorporate automated design of new experiments. First, we use the previously described modeling procedure to generate a library of models corresponding to different gene-regulatory systems in yeast. Many of these models contain transcriptional interactions for which the regulatory effects (inducer versus repressor) are ambiguous and cannot be determined from publicly available expression profiles. Next, to address these ambiguities we implement a score function that ranks possible genetic perturbation experiments on the basis of their projected information content over the models. We perform four of the highest-ranking perturbations experimentally and integrate the data back into the model. The new data support two out of three novel regulatory pathways predicted to mediate expression changes downstream of the yeast transcriptional regulator *SWI4*.

## Results

### Summary of physical regulatory models

We applied a previously described network-modeling procedure [18] to integrate three complementary sources of gene-regulatory information in yeast: 5,558 promoter-binding interactions for 106 transcription factors measured using chromatin immunoprecipitation followed by microarray chip hybridization (ChIP-chip) [3]; the set of all 15,116 pairwise protein-protein interactions recorded in the Database of Interacting Proteins as of April 2004 [19]; and a panel of mRNA expression profiles for 273 individual gene-deletion experiments [20]. Software for performing the network-modeling procedure is available as a plug-in to the Cytoscape package [21,22] on our supplementary website [23].

For each gene-deletion experiment, the modeling procedure identified the most probable paths of protein-protein and promoter-binding interactions that connect the deleted gene (the perturbation) to genes that were differentially expressed in response to the deletion (the effects of perturbation). Thus, a path represented one possible physical explanation by which a deleted gene regulates a second gene downstream. From the expression data, each interaction on a path was annotated with its probable direction of information flow and its probable regulatory effect as an inducer or repressor.

**Figure 1:**
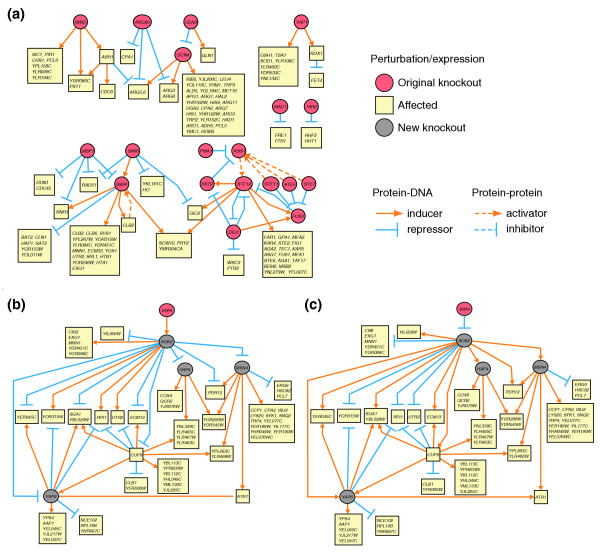
Wiring diagrams for example network models. **(a) **Model 0, showing regulatory pathways that have unique functional annotations. **(b,c) **Model 1, showing regulatory pathways downstream of *SWI4 *and *SOK2 *with ambiguous functional annotations (several would be consistent with the observed expression responses: two possibilities are shown in (b) and (c), respectively). In the models, a connection from gene *a *to *b *represents the experimental observation that the proteins encoded by *a *and *b *physically interact in a protein-protein interaction (dotted links), or that the protein encoded by *a *binds the promoter of *b *(solid links). Each gene is either defined by an original knockout (red nodes), a differentially expressed effect (yellow nodes), or a signal transducer that was chosen for follow-up perturbation (gray nodes). Functional annotations (edge colors) are uniquely determined in (a) whereas multiple annotations are possible in (b) and (c) based on the available data. Diagram layout is performed automatically using the Cytoscape package [21].

For example, the model in Figure 1a (top center) includes a path from *GLN3 *through *GCN4 *to a block of downstream affected genes. This model integrates evidence that: Gln3p binds the promoter of *GCN4 *with high significance in a ChIP-chip assay [3] (*p *≤ 8 × 10^-4^); Gcn4p binds the promoters of many genes in the ChIP-chip assay (*RIB5*, *YJL200C*, and others in the downstream block); and a significant number of genes in the block are upregulated in a *gln3*Δ knockout but downregulated in a *gcn4*Δ  knockout [20]. Together, this evidence confirms Gcn4p as an activator of downstream genes [24] and leads to a (novel) annotation that Gln3p is likely to regulate *GCN4 *via transcriptional repression.

In total, the modeling process generated 4,836 paths, each explaining expression changes for a particular gene in one or more knockout experiments. Of the 965 interactions covered by paths, 194 had regulatory effects that were uniquely determined by the data, while regulatory effects of the remaining 771 interactions were ambiguous. For example, Figure 1b includes ambiguous interaction paths through *SWI4*, *SOK2*, and *MSN4*, explaining the observation that many genes for which the promoters are bound by Msn4p are upregulated in a *swi4*Δ  knockout. This observation can be explained by several alternative annotations: one scenario is that *SWI4 *activates *SOK2 *and *SOK2 *represses *MSN4 *(Figure 1b), whereas another is that *SWI4 *represses *SOK2 *and *SOK2 *activates *MSN4 *(Figure 1c). These regulatory annotations could be uniquely determined by measuring the expression changes of genes downstream of *MSN4 *in the model in response to a *sok2*Δ  deletion and an *msn4*Δ  deletion (see below).

Paths with ambiguous interactions were partitioned into 37 independent network models (numbered 1-37), where each model contained a distinct region of the physical network (see Materials and methods and Additional data file 1). The remaining non-ambiguous paths were grouped into a single model (Model 0). As shown in Table 1, 21 of the models (55%) contained pathways that are well documented in the literature or are significantly enriched for genes belonging to specific Munich Information Center for Protein Sequences (MIPS) [25] functional categories. Of 132 protein-DNA interactions incorporated into Model 0, we found that 50 had been confirmed in classical (low-throughput) assays as reported in the Proteome BioKnowledge Library [26]. Moreover, the inferred regulatory roles (induction or repression) for 48 out of 50 of these interactions agreed with their experimentally determined roles (96%, binomial *p*-value < 1.22 × 10^-7^). Wiring diagrams for Models 0 and 1 are given in Figure 1; diagrams for all other regulatory network models are provided in Additional data file 1 and at [23].

### Experiment selection

**Figure 2:**
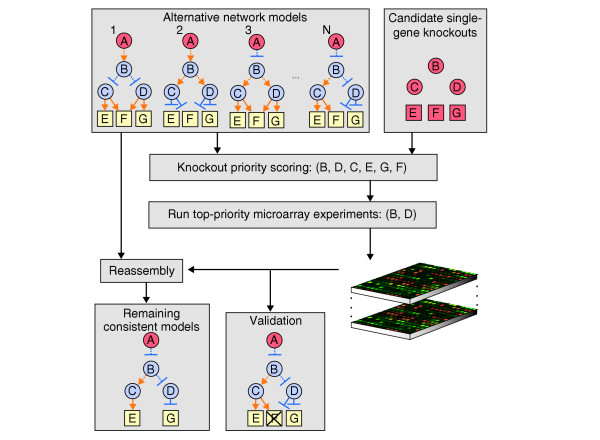
Schematic of the experimental design approach. The input to the approach is a set of alternative representations of a gene-regulatory model, each of which is equally likely given current expression data. In the present work, the alternatives arise as a result of ambiguities in the regulatory roles of interactions in the model as inducers or repressors of downstream genes. Next, a scoring procedure is used to rank candidate perturbations according to their expected information gain over the model alternatives. High-ranking perturbations are applied to the system and characterized using gene-expression microarrays. The resulting expression profiles validate or invalidate particular connections in the model and reduce the set of model alternatives to those that are consistent with both old and new expression measurements.

As shown in Figure 2, we implemented an information-theoretic approach to discriminate between ambiguous model annotations using the fewest additional gene-expression experiments. All non-lethal single-gene knockout experiments were ranked by their projected information content based on the inferred models (see Materials and methods). Table 2 reports the list of top-ranking experiments. This list coincides roughly with biological intuition, in the sense that informative target genes typically encode proteins that are network 'hubs', each having a large number of regulatory interactions with downstream genes in the models. However, as discussed later, knocking out hubs only is not as effective as using the information-theoretic criteria.

Among the highest-priority experiments, Model 1 (Figure 1b) was the most often targeted, containing three of the top 10 highest-scoring genetic perturbations: *sok2*Δ, *yap6*Δ, and *msn4*Δ. A fourth perturbation to Model 1, *hap4*Δ, was also highly ranked (rank 34). Therefore, Model 1 was chosen for further experimentation.

### Model validation

Knockout strains corresponding to the high-ranking perturbations *sok2*Δ, *yap6*Δ, *hap4*Δ, and *msn4*Δ  were grown in quadruplicate under conditions identical to those for the initial 273 knockouts by Hughes *et al. *[20]. Gene-expression profiles were obtained for each knockout culture versus wild type using yeast genome microarrays. We sought to test the three regulatory cascades leading from *SWI4 *to *SOK2 *to either *MSN4*, *HAP4*, or *YAP6 *(Figure 1b). To verify these cascades independently of the model, we analyzed the expression patterns of gene sets known to be directly regulated by *MSN4*, *HAP4*, or *YAP6 *(obtained from the Proteome BioKnowledge Library [26]; see Additional data file 1). To normalize between our microarray procedures and those of Hughes *et al*., we also repeated the original *swi4*Δ  expression profile, and filtered the above sets to select only those genes with expression changes that were reproducible (that is, same direction of change) between the Hughes *et al*. *swi4*Δ  profile and our new profile. Expression changes were reproducible for 28 of 42 Msn4p-regulated genes, 11 of 29 Hap4p-regulated genes, and 64 of 119 Yap6p-regulated genes. Expression similarity among the genes in each filtered set was captured formally in a measure called 'coherence'; details of the computation of expression coherence and the selection of the gene sets are described further in Materials and methods and [23].

**Figure 3:**
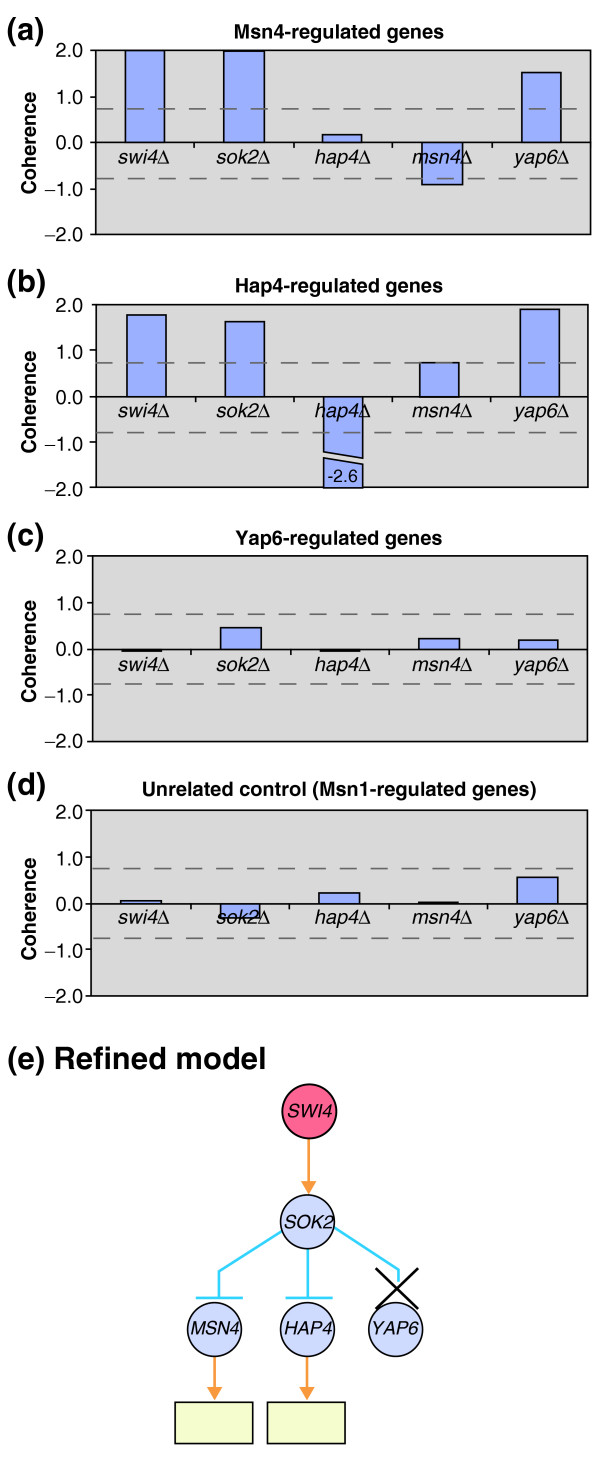
Validation and refinement of Swi4 transcriptional cascades. Yeast genome microarrays were used to explore three transcriptional cascades from Model 1 involving the transcriptional regulators Swi4p, Sok2p, and either **(a) **Msn4p, **(b) **Hap4p, or **(c) **Yap6p. Bar charts show the expression coherence of genes regulated by Msn4p, Hap4p, or Yap6p in knockout strains *swi4*Δ, *sok2*Δ, *msn4*Δ, *hap4*Δ, and *yap6*Δ. Coherence scores more extreme than ± 0.7 are significant (*p *< 0.01, dotted lines). **(d) **Results are also shown for genes bound by Msn1p as representative of an unrelated model not targeted by these perturbations. This analysis provides validation for the Msn4 and Hap4 pathways and disambiguates the role of each pathway interaction as activating (Swi4 interactions) or repressing (Sok2 interactions) downstream genes **(e)**. The Yap6 pathway hypothesis is not supported by this analysis.

As shown in Figure 3a, the gene set downstream of *MSN4 *showed coherent upregulation in the *swi4*Δ  (*p *≤ 10^-4^) and *sok2*Δ  (*p *≤ 10^-4^) knockouts, but downregulation in the *msn4*Δ  (*p *≤ 8 × 10^-4^) knockout. This result supports the existence of a regulatory cascade leading from *SWI4 *to *SOK2 *to *MSN4*. Furthermore, in the context of the present regulatory cascade, *MSN4 *appears to be an inducer as its downstream gene set was downregulated in the *msn4*Δ  experiment. In contrast, *SOK2 *appears to be a repressor of *MSN4 *as a *sok2*Δ  deletion experiment upregulates the same set of genes. Finally, *SWI4 *appears to be an inducer of *SOK2 *as the *swi4*Δ  knockout has the same effect as *sok2*Δ  (that is, upregulation).

Results were qualitatively similar for the *HAP4 *pathway (Figure 3b). The gene set downstream of *HAP4 *was upregulated in the *swi4*Δ  (*p *≤ 10^-2^) and *sok2*Δ  (*p *≤ 9 × 10^-4^) knockouts but downregulated in *hap4*Δ  (*p *≤ 10^-4^). These results suggest that *swi4*Δ, *sok2*Δ, and *hap4*Δ  deletions affect the set of genes immediately downstream of *HAP4*, supporting the *SWI4-SOK2-HAP4 *regulatory pathway hypothesis. In contrast to the *MSN4 *and *HAP4 *pathways, the gene set downstream of *YAP6 *had insignificant responses to all follow-up knockout experiments (Figure 3c). Thus, the existence of the *SWI4-SOK2-YAP6 *regulatory pathway was not supported by our validation experiments.

### Automated model refinement

We used our modeling procedure to construct a new physical network model using the original 273 knockout gene-expression experiments of Hughes *et al*. combined with the new *sok2*Δ, *hap4*Δ, *msn4*Δ, and *yap6*Δ  profiles. Overall, 60 protein-DNA interactions were disambiguated by our data: 50 interactions were resolved as definite inducers or repressors, whereas ten interactions were removed from the model because the expression of downstream genes did not change as a result of the knockout. In the updated Model 1, *MSN4 *and *HAP4 *were unambiguously annotated as inducers of downstream genes, *SOK2 *was annotated as a repressor of *MSN4 *and *HAP4*, and *SWI4 *was annotated as an inducer of *SOK2 *(Figure 3e). These results agree with our previous manually derived annotations (see 'Model validation' above).

### Learning-curve analysis

We quantified the efficiency of our information-based approach by comparing it to two other methods of prioritizing gene knockout experiments: prioritizing hubs and prioritizing genes randomly. First, we generated a 'reference' model by fixing each ambiguous interaction in Models 1-37 to be an inducer or repressor. Assignments were chosen arbitrarily from the set of annotations that were consistent with the original knockout data. Next, we used each method (information, hub, or random) to iteratively 'learn' these assignments. In each iteration, we selected the highest-priority knockout experiment, simulated the resulting expression changes (up/down) using the reference model, updated the inferred model, and recorded the number of ambiguous interactions that were resolved. This iterative learning procedure was repeated 100 times.

**Figure 4:**
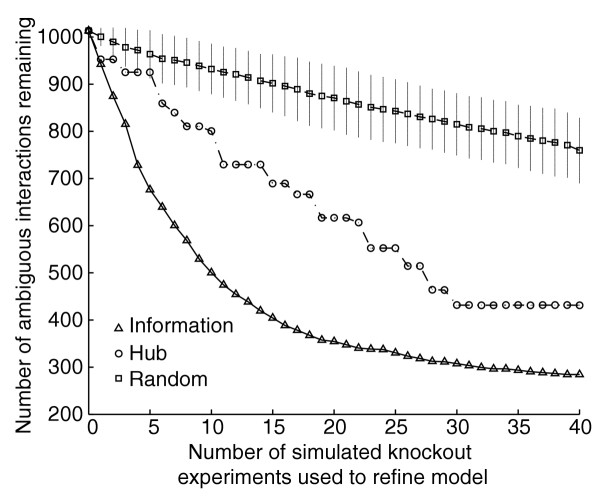
Simulated learning curves of three experimental design methods. Three different methods of selecting experiments are compared: mutual information scores (triangles), hub selection (circles), and random selection (squares). We performed 100 simulated trials and show the average number of ambiguous interactions remaining in the inferred model after each simulated knockout experiment. Vertical bars indicate the standard deviations for the random selection method. The standard deviations for the information and hub selection curves are less than five and are not shown for clarity.

As shown in Figure 4, the mutual information criterion significantly outperformed hub-based and random selection. The learning curves also provide an estimate of the number of additional experiments needed to reduce model ambiguity below a given level. For example, using the information-based score, ten knockout experiments are needed to reduce the number of ambiguous interactions by 50%. In contrast, over 25 experiments are needed according to the hub-based method. Figure 4 suggests that performing 40 additional experiments selected using the information-based score will clarify the regulatory roles of about 70% of the ambiguous interactions. The learning rate of the final 30% becomes very slow because these interactions are isolated in the physical network, unconnected to others, and thus require separate knockouts to decipher each of them.

## Discussion

We have used global expression profiles to validate models of transcriptional regulation inferred from protein-protein interactions, genome-wide location analysis, and expression data. A previously described network inference algorithm [18] identifies probable paths of physical interactions connecting a gene knockout to genes that are differentially expressed as a result of that knockout. The proposed validation strategy uses information gain as a criterion for choosing optimal knockouts to profile using microarray experiments. This strategy agrees with intuition, in that optimal knockouts typically target intermediate genes along the pathways under consideration. If an intermediate gene knockout fails to affect downstream genes in a pathway, that pathway is removed from the model.

The validated pathways point to a combination of previously documented and novel findings. First, in agreement with previous literature, we confirm that *MSN4 *and *HAP4 *are inducers [27,28] and that *SOK2 *is a repressor [29]. For instance, *SOK2 *is known to act downstream of protein kinase A (PKA) to repress genes involved in stress response, glycogen storage, and pseudohyphal growth [29]. However, although *SOK2 *is thought to control these pathways via a transcriptional cascade, the components of this cascade have remained unclear. Here, we provide evidence for a model in which *SOK2 *acts as a negative regulator upstream of *MSN4 *and *HAP4*. Interestingly, *MSN4 *has been shown to activate stress-response genes [28], and *HAP4 *has been shown to activate genes involved in energy conservation and oxidative carbohydrate metabolism [27]. Thus, we have identified a candidate model for the transcriptional cascade downstream of PKA signaling that mediates stress response. This model includes two novel regulatory pathways from *SWI4 *to *SOK2 *to *MSN4 *and from *SWI4 *to *SOK2 *to *HAP4*. The validation experiments do not support the third predicted pathway from *SWI4 *to *SOK2 *to *YAP6*.

In model simulations, choosing new gene knockout experiments with an information-theoretic approach significantly outperformed both random and hub-based selection. It also outperformed the observed experimental results: approximately 280 interactions were disambiguated after four simulated knockouts (Figure 4), whereas only 60 interactions were resolved due to the four actual knockouts *sok2*Δ, *hap4*Δ, *msn4*Δ, and *yap6*Δ. This difference in performance stems from key differences between the simulated and actual scenarios. In simulation, the four experiments are performed independently and iteratively, selecting the absolute highest-ranking knockout each time. In the actual study, four high-ranking experiments (but not the highest) are chosen to interrogate and maximally resolve a single pathway model, resulting in experiments that are highly co-dependent and performed simultaneously without intervening rounds of inference and experimental design. In addition, the simulation assumes that all interactions in the model are correct, along with one of the initial sets of inducer/repressor annotations. It therefore isolates the process of learning regulatory role annotations, whereas the actual procedure also serves to distinguish interactions as true versus false positives. Nevertheless, the simulation provides a useful comparison of experimental design methods relative to each other.

An important limitation of the single-gene knockout approach is that single perturbations do not identify pathway intermediates for which loss of function can be compensated by another gene. Furthermore, our approach may not identify regulatory pathways in which several transcription factors independently activate gene expression. Applying knockouts in combination may prove fruitful in these cases. For instance, approximately 4,000 double knockouts have been reported in yeast that lead to synthetic lethality: that is, a lethal phenotype that is not observed in either of the single knockouts individually [30]. These interactions suggest regulatory relationships which could be incorporated into future work.

## Conclusion

Scientific discovery is an iterative process of building models to explain experimental observations and validating models with new experiments [31]. Experimental design is the essential link between these two aspects. Here we have explored a framework for modeling transcriptional networks in which experimental design and validation are central features. This framework is based on computational analysis and expression microarrays, both of which are amenable to automation, suggesting a high-throughput strategy for mapping gene-regulatory pathways.

## Materials and methods

### Model building and inference

Physical mechanisms of transcriptional regulation were modeled using an approach described previously [18]. Briefly, we postulated that the regulatory effects of deleting a gene are propagated along paths of physical interactions (protein-protein and protein-DNA). We formalized the properties of these paths and interactions using a factor graph [32] and found the most probable set of paths using the max-product algorithm [32]. The resulting set of paths was partitioned into independent network models, also as described previously [18]. The raw data used in the modeling procedure included 5,558 promoter-binding interactions (at *p*-value < 0.001) for 106 transcription factors [3], the set of all 15,166 pairwise protein-protein interactions recorded in the Database of Interacting Proteins as of April 2004 [19], and mRNA expression profiles for 273 individual gene deletion experiments [20]. Expression changes with a *p*-value < 0.02 were considered significant.

### Experiment scoring

We calculated the expected information gain for each of the 4,756 possible non-lethal single-gene deletion experiments that were not included in the set of 273 deletions used to generate our network models. Intuitively, information gain measures (the logarithm of) the number of ambiguous annotations in the model that are likely to be determined after generating a yeast-genome expression profile in response to a particular gene deletion under consideration. Each gene-deletion experiment predicts a distinct expression profile given a particular configuration of model annotations. Experiments with high information gain are those for which the predicted expression profiles are highly variable over the set of possible annotations. In these cases, only one (or at most a few) of the predicted profiles will match the true observed profile, efficiently constraining the space of possible model annotations.

The information gain discussed above arises from the expected value of information calculations in statistical decision theory [12]. Here we describe the score more directly in terms of reduction of model entropy. The entropy of a set of ambiguous model annotations is given by:



The expected information gain is the difference between the entropies before and after a hypothetical experiment:



where *Y*^*e *^denotes the vector of predicted expression changes for each gene in the model under experiment *e*. The conditional entropy *H*(*M*|*Y*^*e*^) requires us to consider all possible models and corresponding outcomes resulting from experiment *e*. Direct enumeration of all values of *M *and *Y*^*e *^is impractical; instead, we make several simplifying approximations as described at [23].

### Expression profiling

Expression profiling experiments were based on the wild-type diploid BY4743 and homozygous gene knockout strains derived from this parent [33] (Invitrogen), with cultures grown identically to those of Hughes *et al*. [20]. Labeled cDNA from each gene knockout strain was co-hybridized versus wild type cDNA in quadruplicate two-color microarray hybridizations. Total RNA was isolated by hot acid phenol extraction, purified to mRNA (Ambion PolyAPure kits), and labeled with Cy3 or Cy5 by direct incorporation (Amersham CyScribe First-Strand cDNA Labeling Kit). DNA microarrays were spotted from the Yeast Genome Oligo Set v1.1 (Qiagen) on Corning UltraGAPS slides using an OmniGrid 100 robot (Genomic Solutions). Lyophilized Cy3- and Cy5-labeled samples were resuspended in 50 μl buffer (5× SSC, 0.1% SDS, 1× Denhardt's solution, 25% formamide) and co-hybridized at 42°C beneath a coverslip for 15 h. Arrays were imaged at 10 μm resolution using a ScanArray Lite instrument (PerkinElmer). Raw quantitated background intensities were smoothed using a 7 × 7 median filter, separately for the Cy3 and Cy5 channels, and data were corrected for cyanine-dye dependent bias using a Qspline normalization [34]. The VERA/SAM package [34] was used to assign a log-likelihood statistic λ with each gene, indicating its significance of differential expression in each experiment. Microarray expression data are deposited in the ArrayExpress database [35] under accession numbers A-MEXP-217 (Arrays) and E-MEXP-351 (Experiments).

### Expression coherence

The expression coherence of a set of genes measures whether the expression levels of these genes behave similarly in a particular experiment. Each gene *i *in gene-deletion experiment *e *has an expression ratio *r*_*ie *_(versus wild type) and associated *p*-value *p*_*ie *_of differential expression. First, we filter out insignificant expression changes with a *p*-value > 0.5. Then, we use the inverse Gaussian cumulative distribution function, Φ^-1^, to convert each remaining *p*-value into a z-score [36,37]:

*z*_*ie *_= Φ^-1 ^(1 - *p*_*ie*_)

Next, we compute a 'signed z-score' by multiplying z by +1 if the expression level is increasing and by -1 if it is decreasing. The average signed z-score for a gene subset of size *N *is computed as:



Gene sets with expression changes that are significant and in the same direction result in large *Z*-values. A distribution of *Z *values obtained from random gene sets of size *N *was used to determine a *p*-value for each expression coherence score.

## Additional data files

Additonal data is available with the online version of this paper. Additional data file 1 contains Tables S1-S4 and wiring diagram illustrations for Models 0-44. Table S1 gives the internal validation for 17 out of 24 restricted network models; Table S2 lists the correlations between swi4δ and gcn4δ data and Rosetta and the new experiments; Table S3 gives the restricted subsets used to evaluate the reproducibility; and Table S4 gives the gene sets for external validation.

## Supplementary Material

Additional File 1 Table S1 gives the internal validation for 17 out of 24 restricted network models; Table S2 lists the correlations between swi4δ and gcn4δ data and Rosetta and the new experiments; Table S3 gives the restricted subsets used to evaluate the reproducibility; and Table S4 gives the gene sets for external validation. Click here for file

## Figures and Tables

**Table 1:** Internal validation for 21 of the 38 inferred models

Model	Number of genes	Number of variants	Validated literature pathway	Enriched MIPS functions
0	130	1	Kss1/Fus3-Ste12 (mating response and filamentous growth)	Cell fate (1.48 × 10-7); metabolism (0.0067)
1	69	8	Sok2-Msn4 (PKA pathway)	
2	63	16	Tup1-Hhf1 (histone regulation)	Protein synthesis (7.13 × 10-8)
3	44	2	Tup1/Ssn6-Nrg1 (glucose metabolism)	Transport (1.05 x 10.5); metabolism (5.41 × 10-4)
4	58	8	Tup1/Ssn6-α2/Mcm1 (mating response)	Cell fate (1.12 × 10-5);
5	58	4	Rpd3-Abf1 (histone modification)	
6	44	2	Swi4-Ndd1-Ace2 (cell cycle)	
7	26	4		Cell cycle (0.035)
8	36	8	Slt2-Rlm1/Swi4 (PKC pathway)	
10	45	16	Med2-Gal4/Gcn4 (general transcription)	
15	13	4	Cmd1-Cna1-Skn7 (calcium signaling)	
19	9	4		Cell defense (6.33 × 10-6)
23	17	2		Metabolism (1.49 × 10-6);energy (0.04)
26	8	8		Cell defense (9.62 × 10-5)
29	9	4	Yap1-Cad1 (metal response)	
30	12	4	Med2-Srb6-Gal4 (general transcription)	
32	12	4	Med2-Gal11-Gal4 (general transcription)	
33	12	4	Med2-Srb5-Gal4 (general transcription)	
34	9	4	Ste12-Mcm1 (mating response)	Cell fate (4.55 × 10-8); homeostasis (0.0012); cell communication (0.0345)
36	7	4		Metabolism (0.0258)
40	5	2		Metabolism (0.0017)

The number of genes and variants are shown for each model along with the results of our preliminary validations. Each variant corresponds to a distinct set of functional annotations on the interactions in the model (directions and regulatory effects, see text). For Model 0, the expression data implied a unique set of annotations; for all other models multiple sets of annotations were possible. Each model was validated if its pathways were (wholly or partially) cited in previous studies or its downstream genes were significantly enriched for MIPS functional categories (*p *≤ 0.05; hypergeometric test with Bonferroni correction).

**Table 2:** Top-ranking knock-out experiments proposed for model discrimination

Gene	Function	Score	Downstream genes	Rank	Model
*HHF1*	Histone	52.1429	74	1	2
SOK2*	Regulator for meiosis and PKA pathway	45.0279	64	2	1
*CKA1*	Protein kinase of cell cycle	45.0075	64	3	5
*A2*	Mating response	40.9023	58	4	4
YAP6*	Stress response regulator	35.1652	50	5	1, 3
*NRG1*	Regulator of glucose dependent genes	31.6501	45	6	3
*FKH1*	Regulator of cell cycle	29.1194	41	7	2
*FKH2*	Regulator of cell cycle	26.7131	38	8	7
*SLT2*	Protein kinase of cell wall integrity pathway	23.4727	31	9	8
MSN4*	Regulator of stress response	21.8224	31	10	1
HAP4*	Regulator of cellular respiration	6.3310	9	34	1

Each proposed target gene is reported, along with its function, mutual information score, rank, and the model(s) it informs. All target genes are non-lethal in rich media. *Gene knockouts selected in this study.
