# Supplementary material for: Validation and refinement of gene-regulatory pathways on a network of physical interactions
Source: Genome Biol. 2005 Jul 1;6(7):R62. doi: 10.1186/gb-2005-6-7-r62 (PMC1175993; doi:10.1186/gb-2005-6-7-r62)

Table S1: Internal validation for 17 out of 24 restricted network models.

Model	# Genes	Validated literature pathway	Refs.	Enriched MIPS Function
0	109	Kss1/Fus3-Ste12 (mating response and filamentous growth)	Elion et al. 1993	cell fate ($1.48x10^{-7}$); metabolism (0.0067)
1	69	Sok2-Msn4 (PKA pathway)	Smith et al. 1998	
2	44	Tup1/Ssn6-Nrg1 (glucose metabolism)	Park et al. 1999	transport ($1.05x10^{-5}$); metabolism ($5.41x10^{-4}$)
3	58	Tup1/Ssn6-α2/Mcm1 (mating response)	Wahi et al. 1995	cell fate ($1.12x10^{-5}$)
4	44	Swi4-Ndd1-Ace2 (cell cycle)	Lew et al. 2001	
6	36	Slt2-Rlm1/Swi4 (PKC pathway)	Jung et al. 1999	
7	13	Cmd1-Cna1-Skn7 (calcium signaling)	Williams et al. 2001	
8	9			cell defense ($6.33x10^{-6}$)
10	12	Med2-Gal11-Gal4 (general transcription)	Park et al. 2000	
12	7			metabolism (0.026)
13	17			metabolism ($1.49x10^{-5}$)
14	6	Gcn4-Met4 (methionine synthesis)		metabolism ($2.88x10^{-4}$)
16	9	Ste12-Mcm1 (mating response)		cell fate ($4.55x10^{-8}$); homeostasis (0.0012); cell communication (0.0345)
17	13	Cka2-Abf1 (casein kinase pathway)		
20	5			metabolism (0.0017)
22	8	Cka2-Abf1 (casein kinase pathway)		
25	7	Cka2-Abf1 (casein kinase pathway)		

Table S2: Correlations between *swi4Δ* and *gcn4Δ* data from Rosetta and the new experiments.

	***swi4Δ* total**	***gcn4Δ* total**
**correlation**	-0.013	0.34
**correlation p-value**	0.84	< 10-4
**rank correlation**	-0.058	0.145
**rank correlation p-value**	1.0	< 10-4
**hyper-geometric p-value**	0.188	4.88×10-21
	***swi4Δ* subset**	***gcn4Δ* subset**
**correlation**	0.344	0.831
**correlation p-value**	8×10-5	< 10-5
**rank correlation**	0.353	0.705
**rank correlation p-value**	9×10-5	< 10-5
**hyper-geometric p-value**	1.05×10-5	< 1.8×10-13

Table S3: Restricted subsets used to evaluate the reproducibility.

Swi4 subset

AAP1	LYS20	YAP6	YJL225C
ATR1	MNN1	YBL029W	YJR078W
BAT2	MSN4	YBL112C	YKL051W
CCP1	NCE102	YBL113C	YLR084C
CLB1	PCL7	YDR451C	YLR463C
CLB2	PDR12	YDR545W	YLR465C
CLB6	PRY2	YEL045C	YLR466W
COX9	QCR2	YEL047C	YLR467W
CPA2	RNR1	YEL077C	YML133C
CSI2	RPI1	YER045C	YNL339C
CUP9	RPL19B	YER189W	YNR067C
ECM13	SAT2	YER190W	YOL011W
ECM33	SCW10	YFR006W	YOR248W
ERG6	SGA1	YGR086C	YOR315W
EXG1	SNQ2	YGR153W	YOX1
HAP1	SOK2	YGR296W	YPL267W
HAP4	SRL1	YHL049C	YPL283C
HSC82	SVS1	YHR048W	YPR203W
HTA1	SWI4	YIL056W	YPS4
HTB1	TRP4	YIL177C	
ISU2	UTR2	YJL217W	

Gcn4 subset

ADH5	HAD1	UGA3
APG1	HAL2	YER069W
ARG1	HIS1	YER073W
ARG11	HIS4	YGL184C
ARG3	HOM3	YHM1
ARG7	LEU4	YHR122W
ARG8	MET16	YHR162W
ARO1	PCL5	YJL200C
ARO3	RIB5	YLR152C
CPA2	TRP2	YMC1
GCN4	TRP3	YOL119C

Table S4: Gene sets for external validation.

Msn4 subset

ALD3	LAP4
ARA1	PGM2
CTT1	RAS2
YMR173W	SOD2
DOT5	SOL4
GLK1	SPS100
GLO1	SSA4
GRE3	TKL2
GSY1	TPS1
HOR2	TTR1
HSP12	YDL124W
HSP26	YKR011C
HSP104	YMR315W
HXK1	YNL200C

Hap4 subset

ATP3	COX13
ATP7	QCR7
ATP16	TUF1
COX4	QCR10
COX7	YLR294C
COX12	

Wiring diagrams for all inferred physical regulatory models

Note: Model 0 contains interactions for which the annotations (activator/repressor) could be uniquely determined. Models 1-44 (38 total) contain ambiguous interactions. Hence, we show only one of many possible sets of annotations that are consistent with the original knockout data.

Model 0

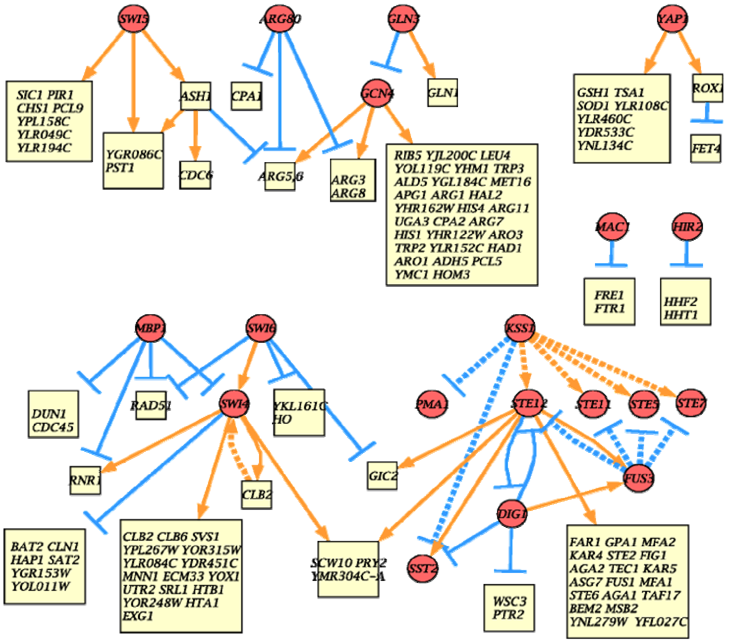

Model 1

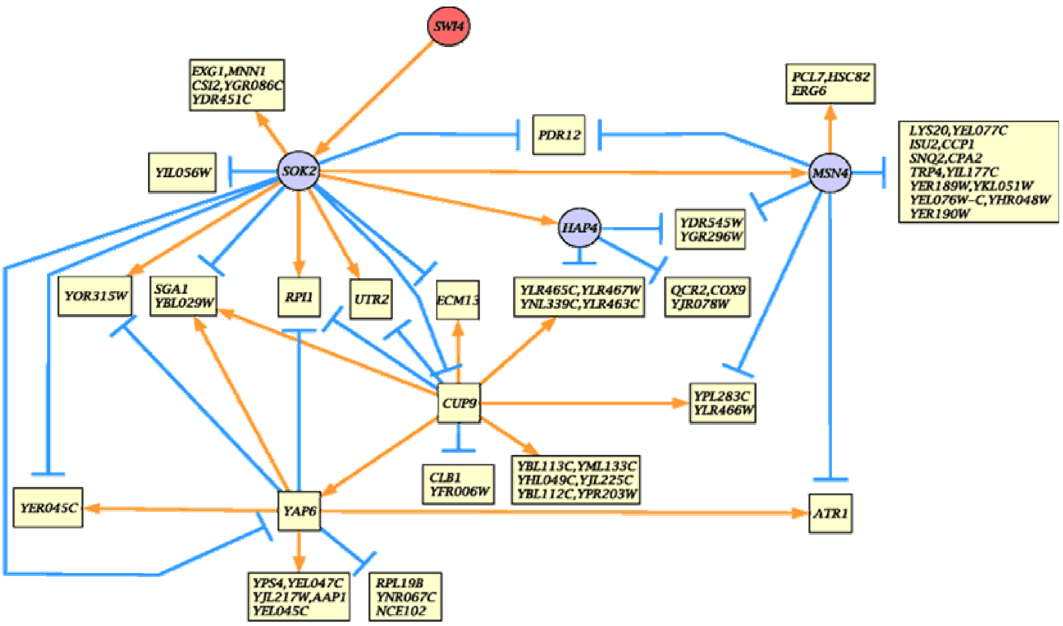

Model 2

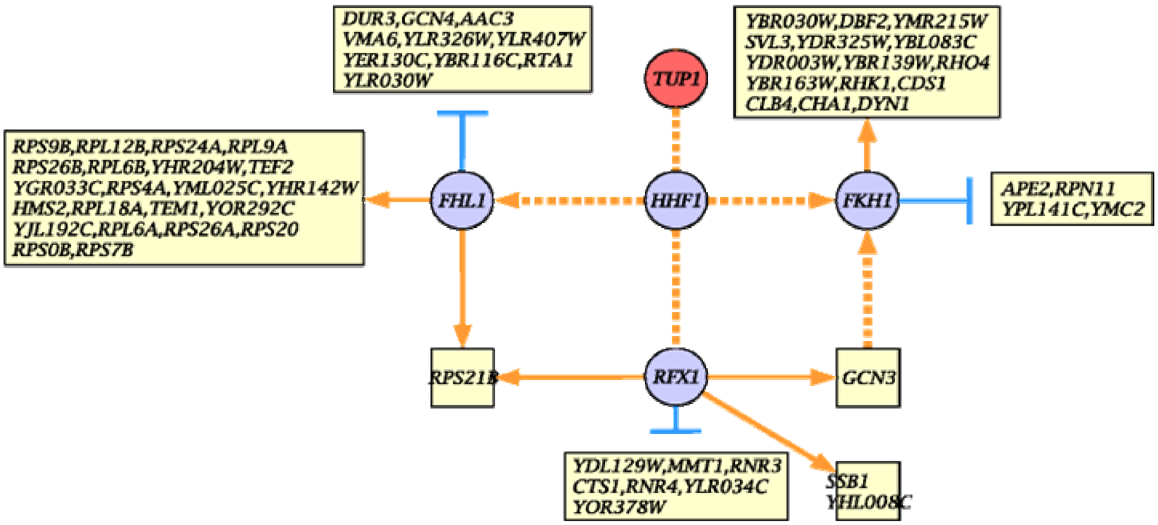

Model 3

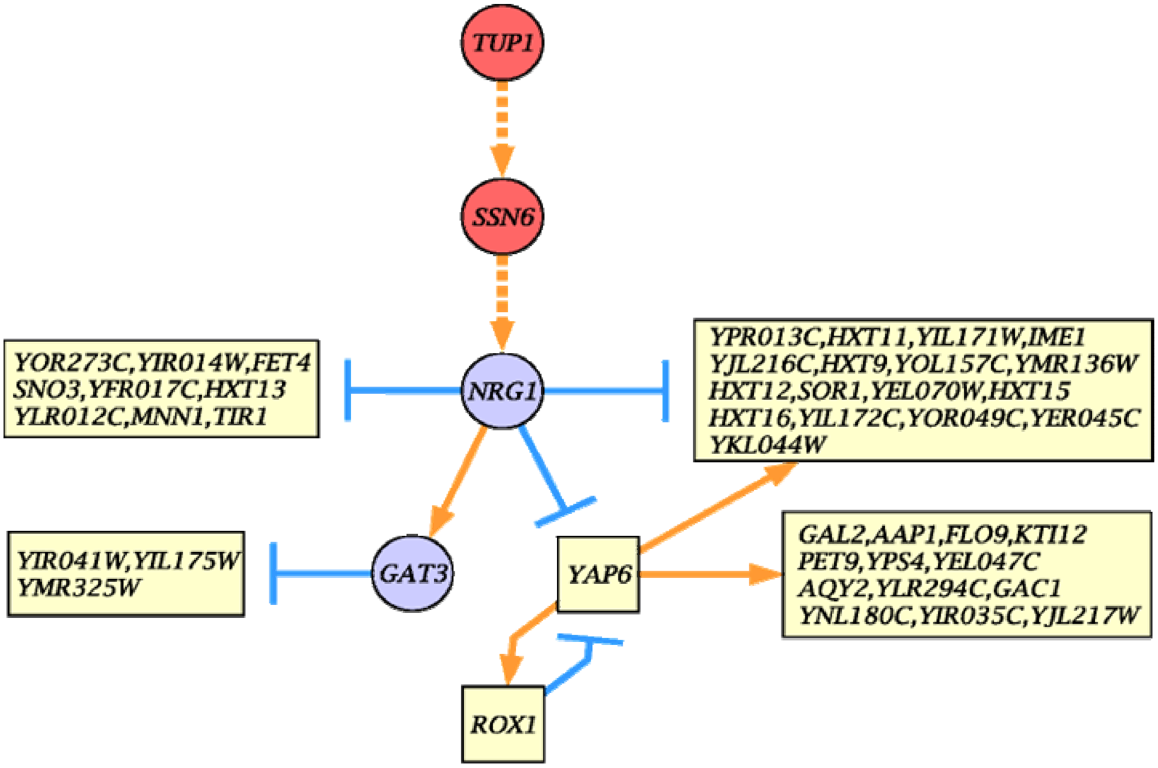

Model 4

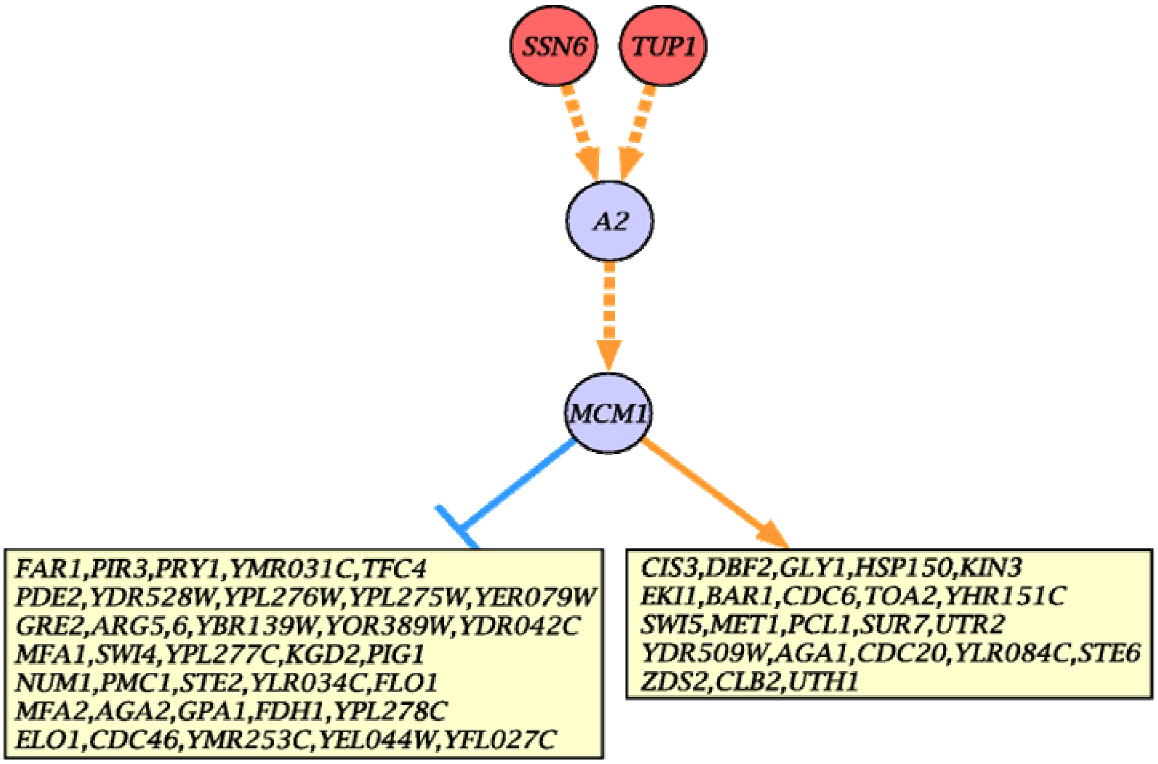

Model 5

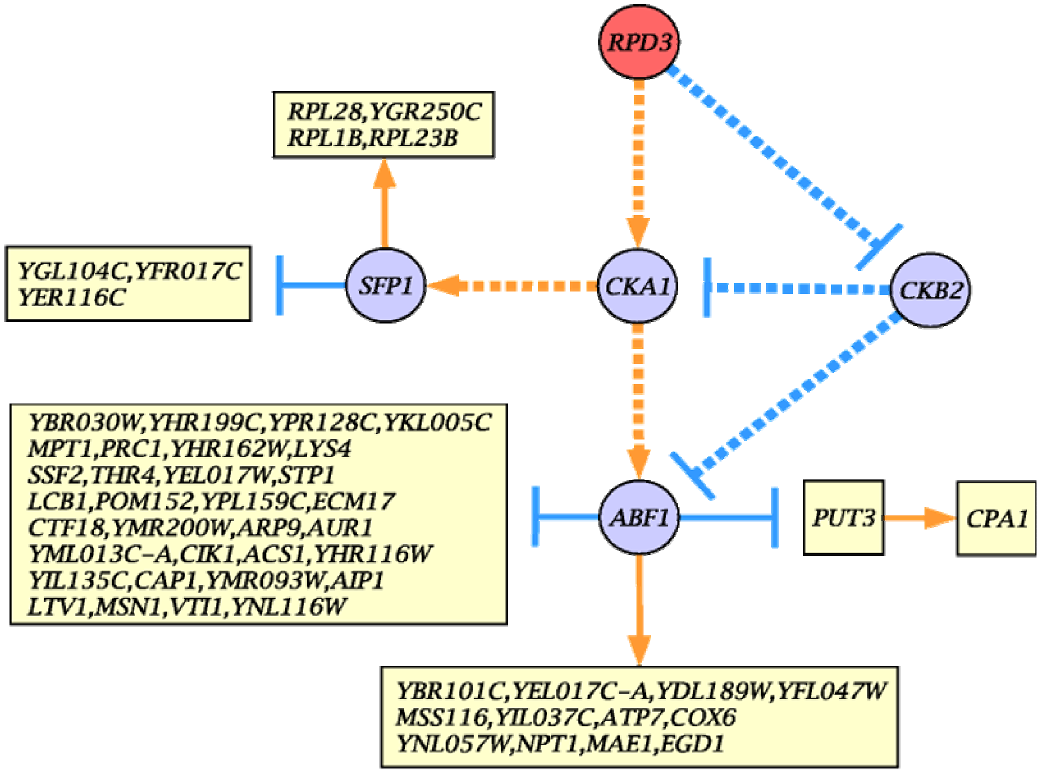

Model 6

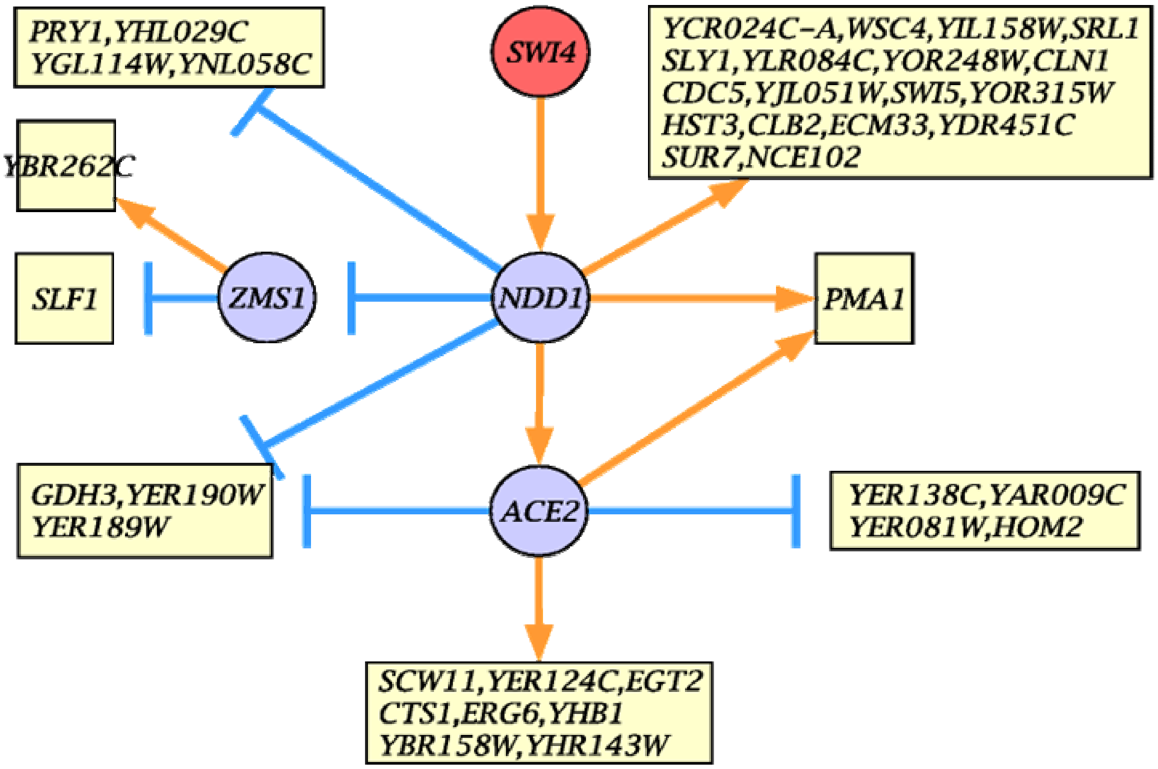

Model 7

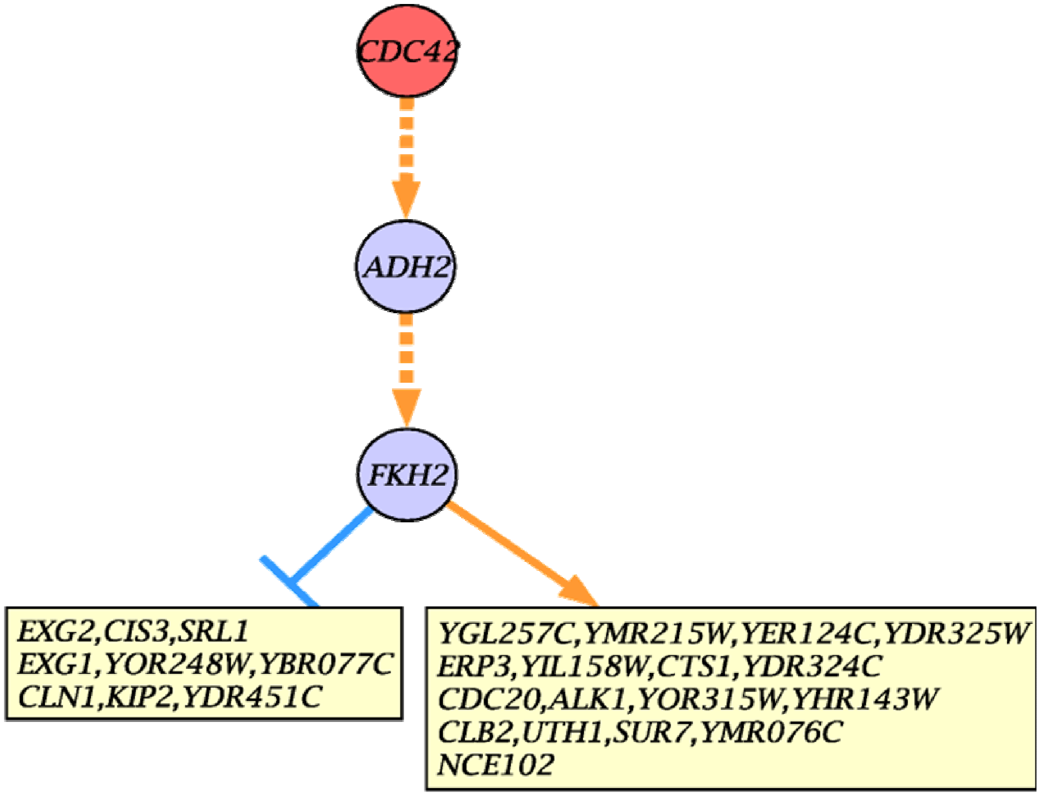

Model 8

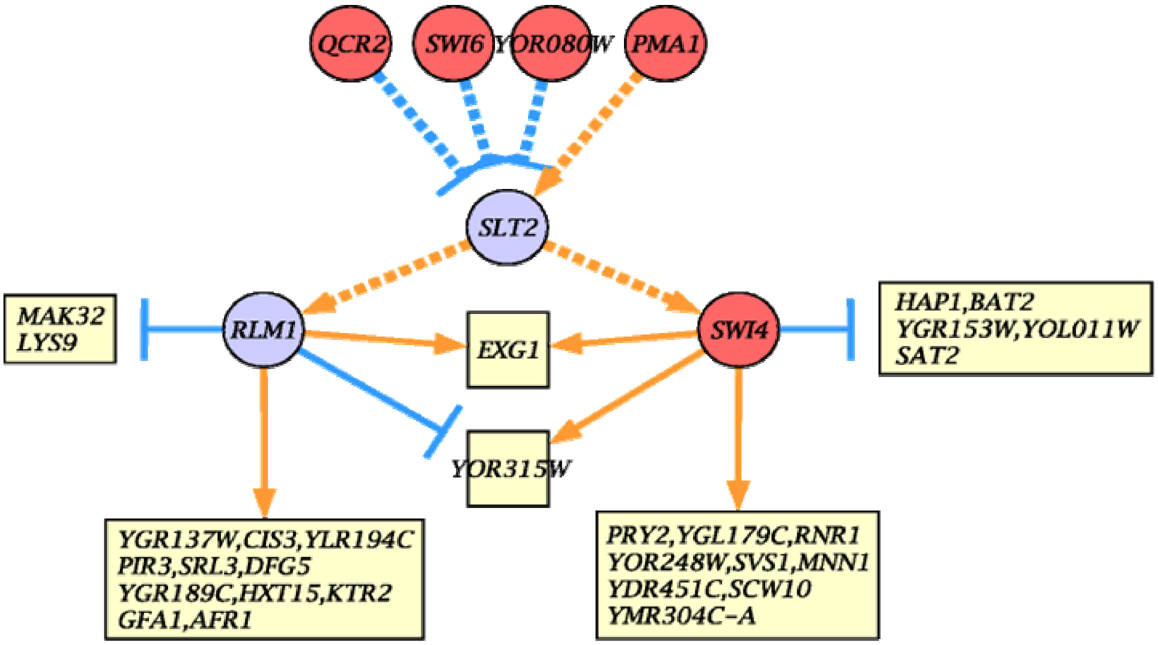

\Model 10

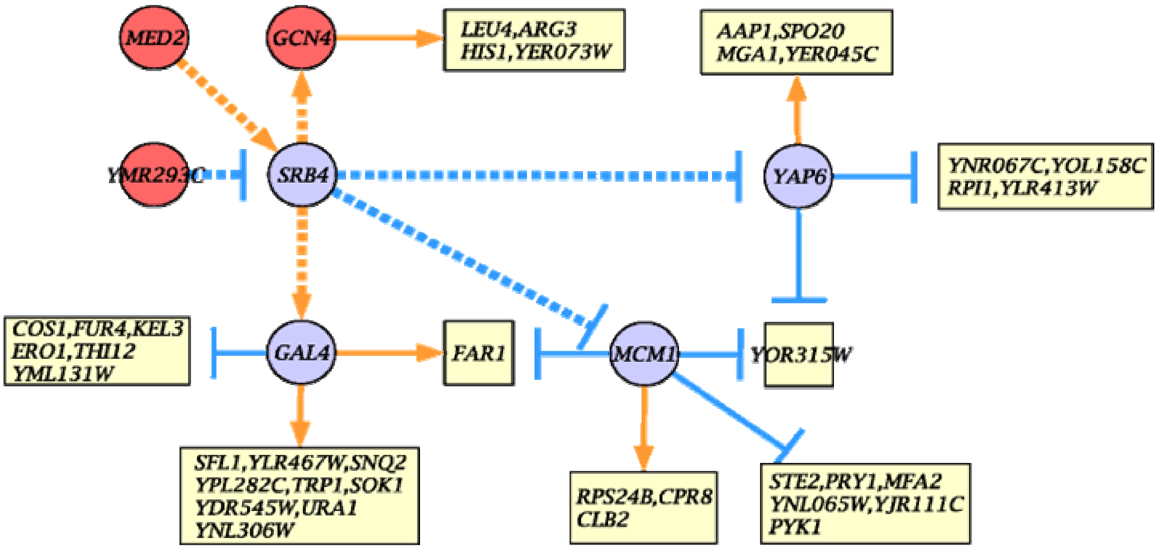

Model 11

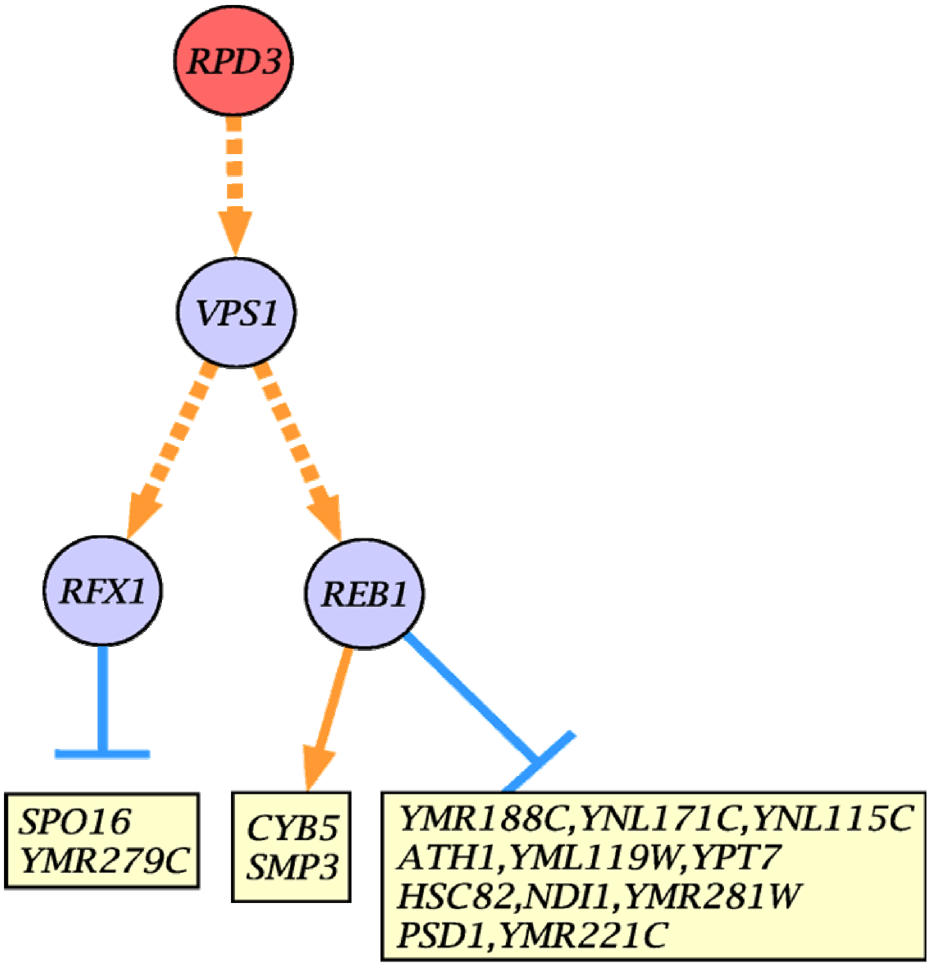

Model 13

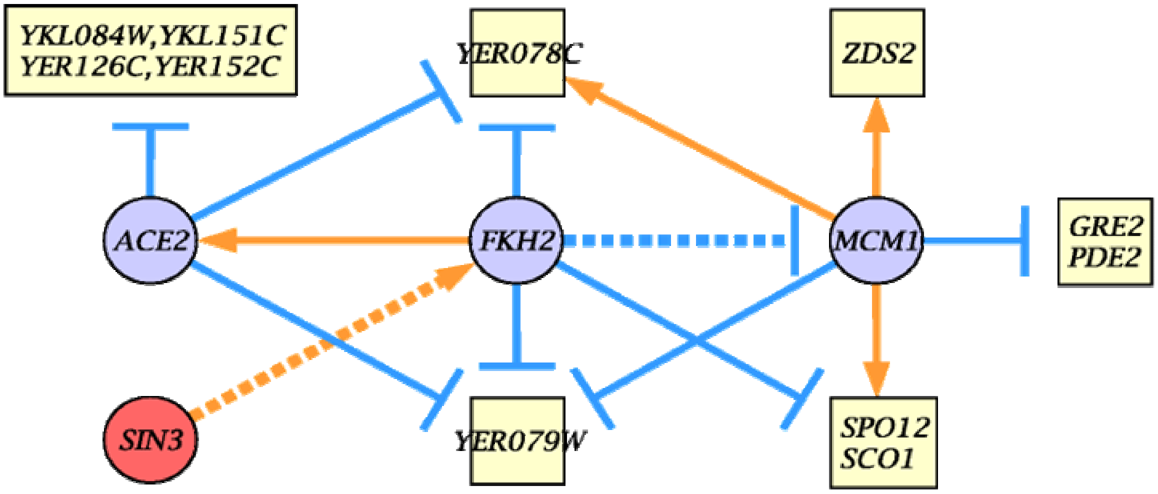

Model 14

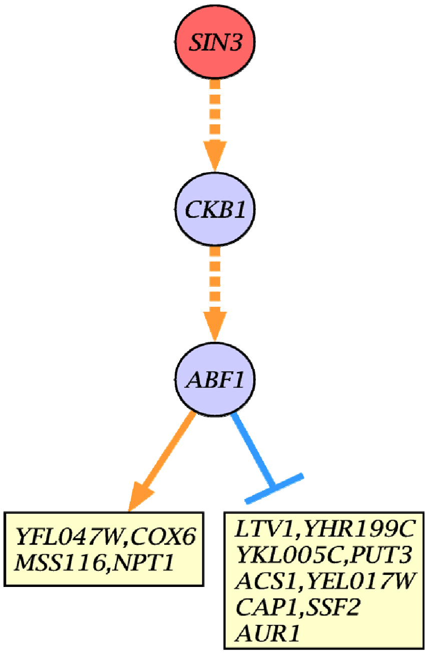

Model 15

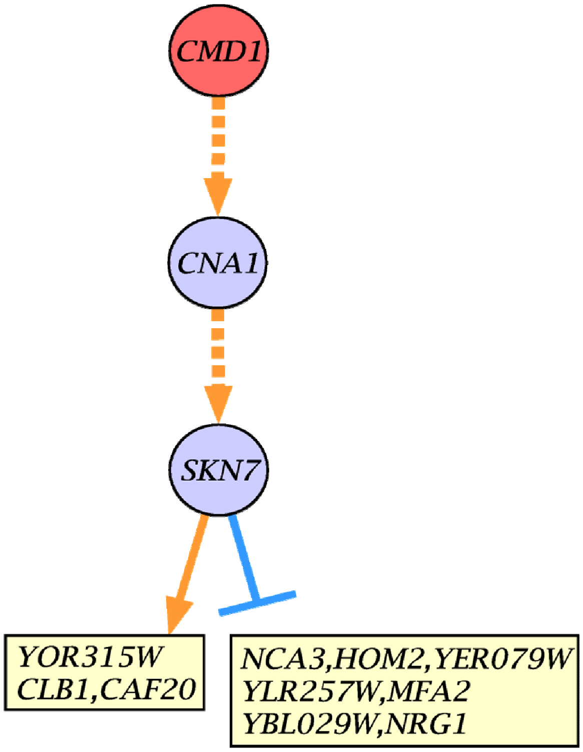

Model 16

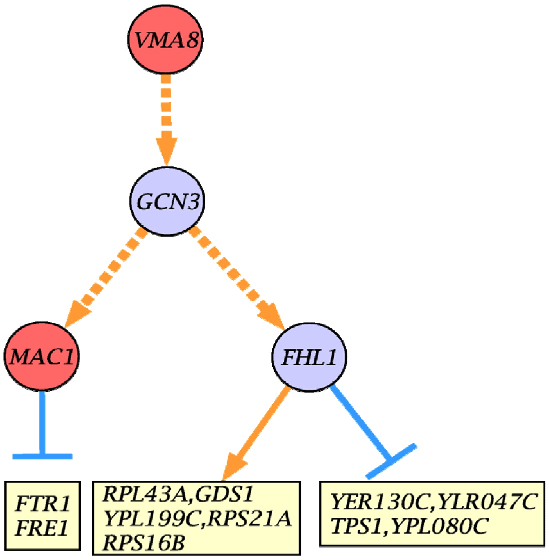

Model 19

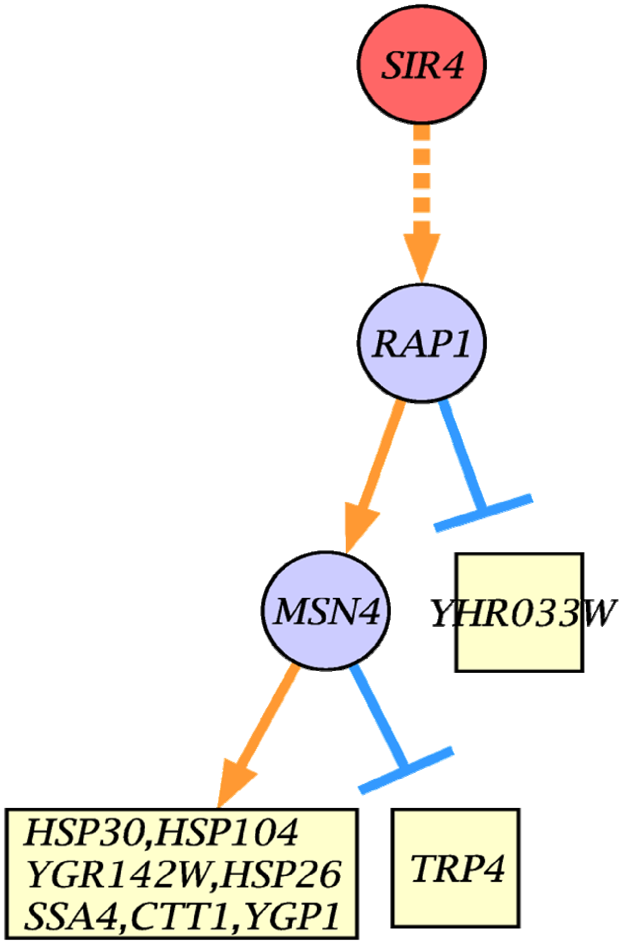

Model 20

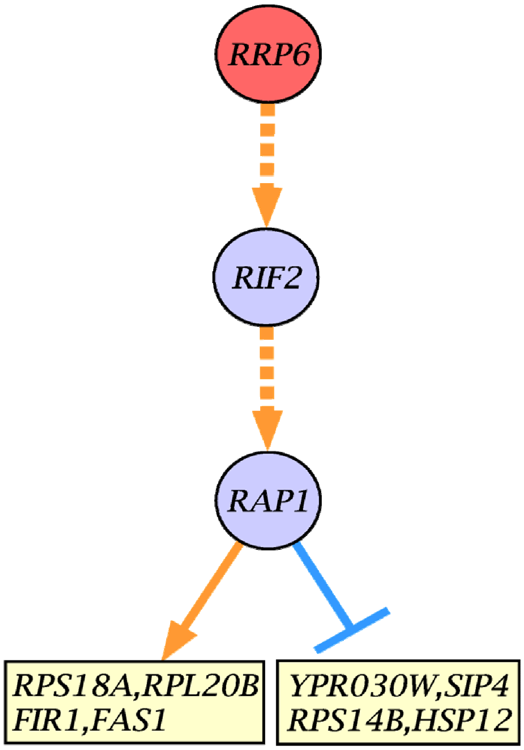

Model 21

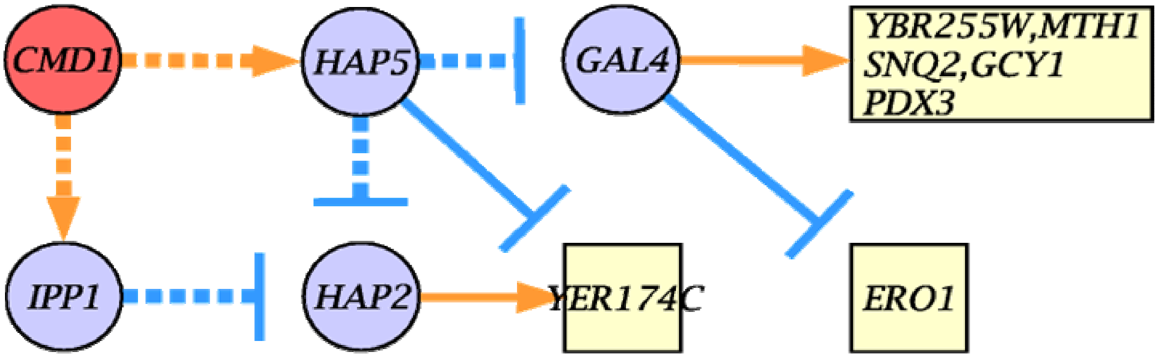

Model 22

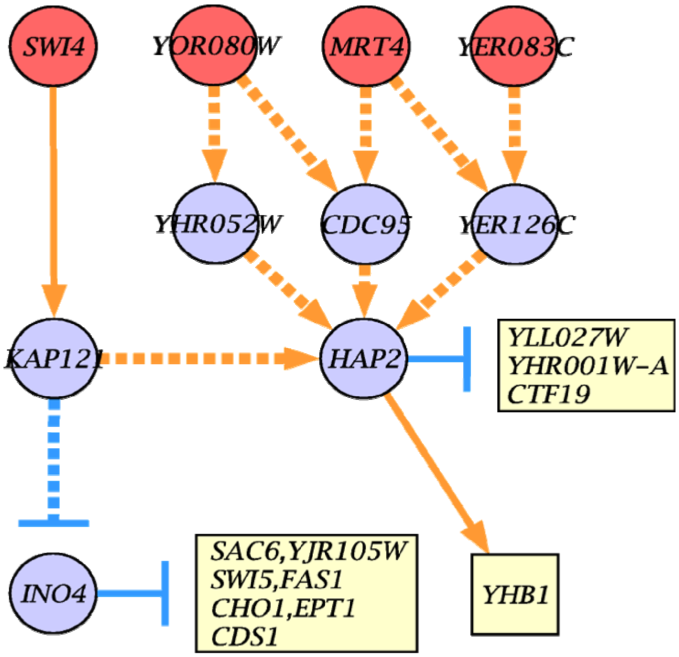

Model 23

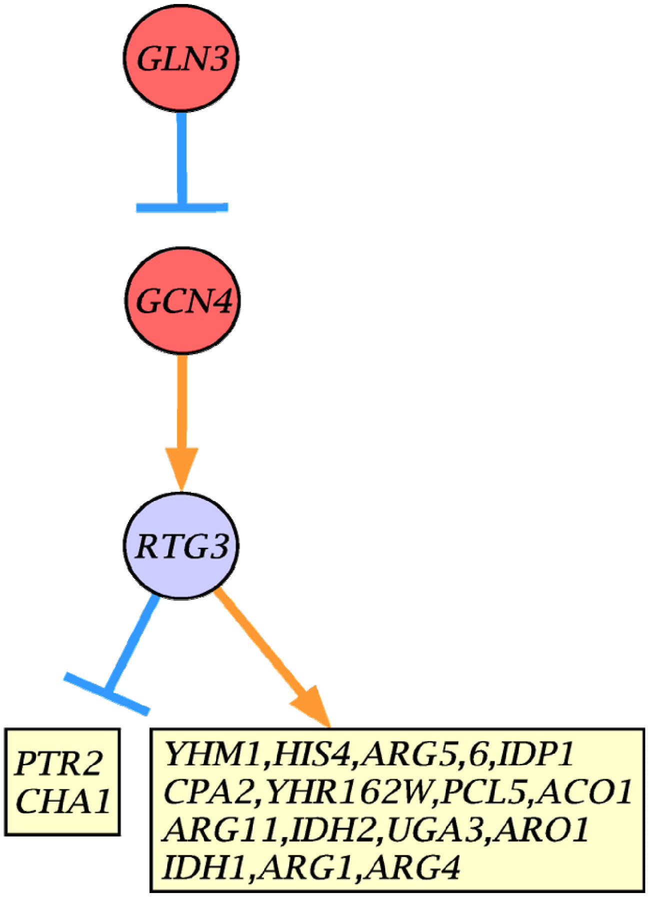

Model 24

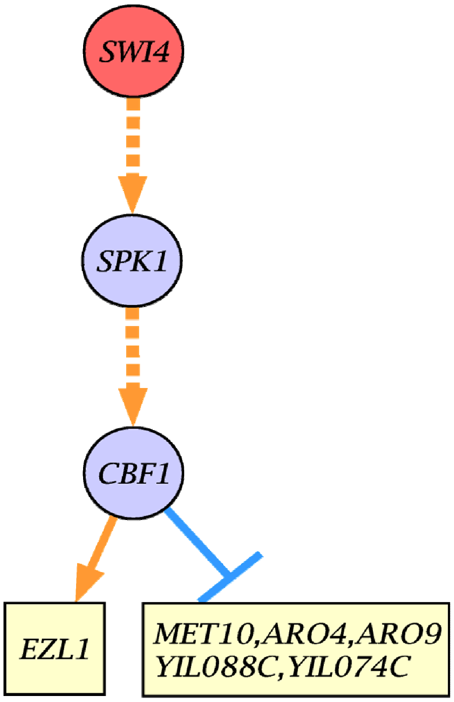

Model 25

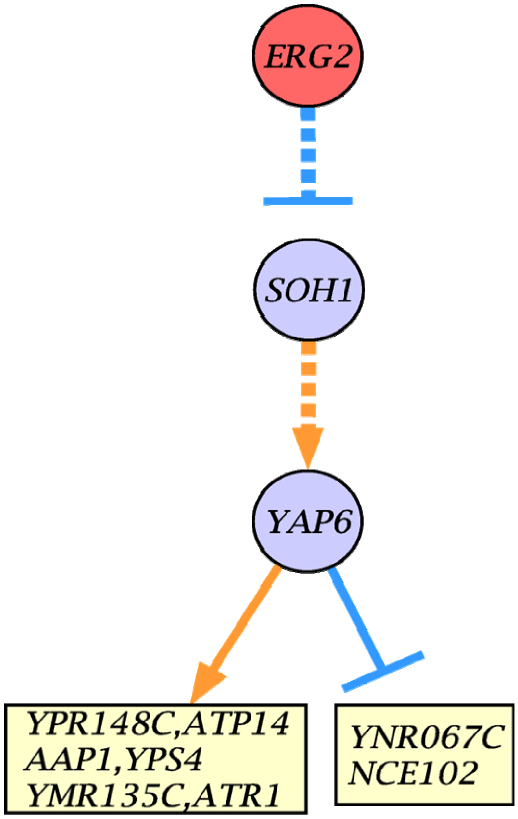

Model 26

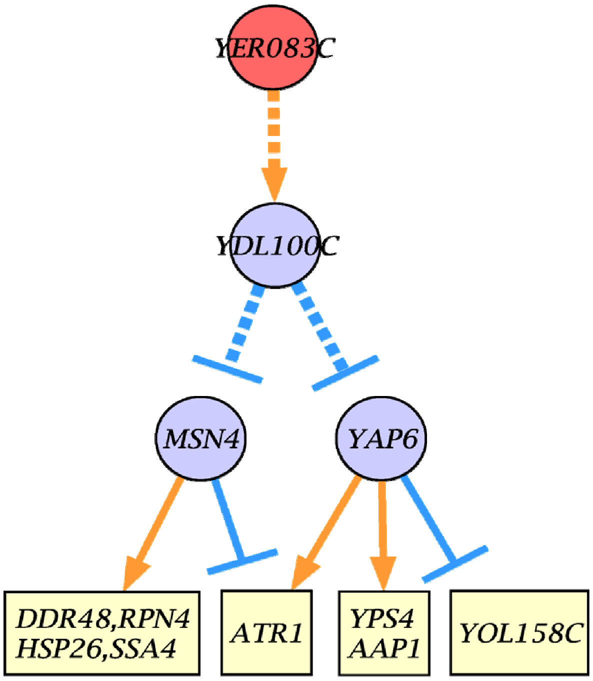

Model 27

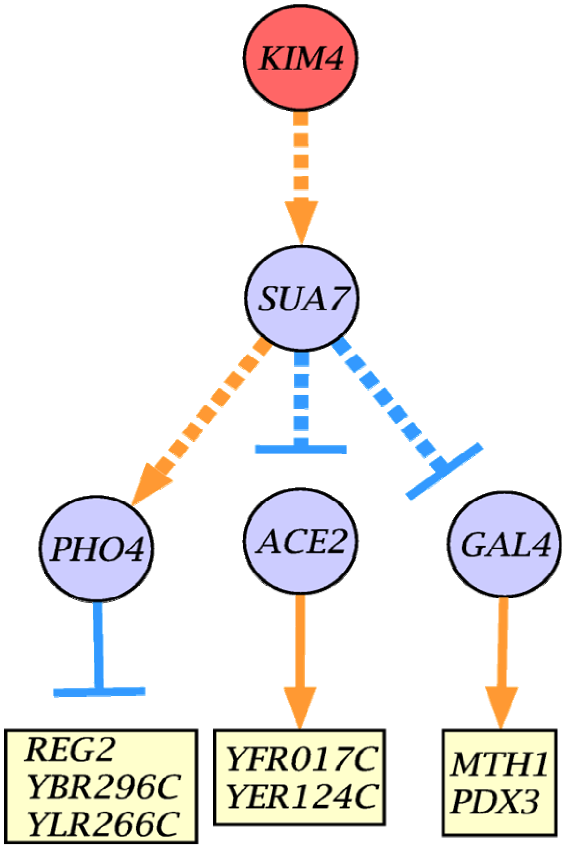

Model 28

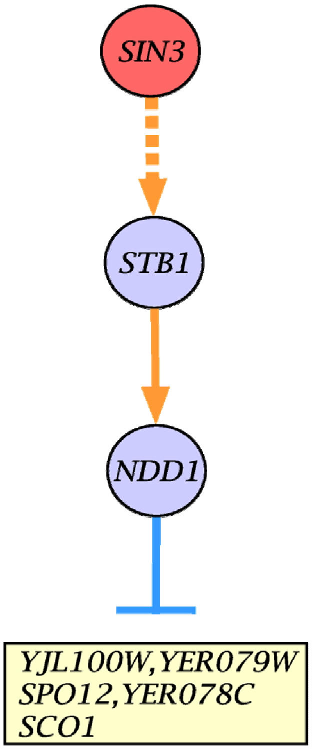

Model 29

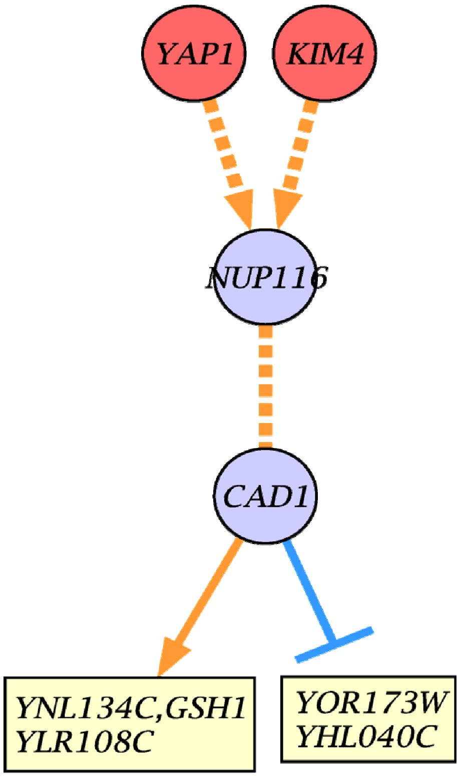

Model 30

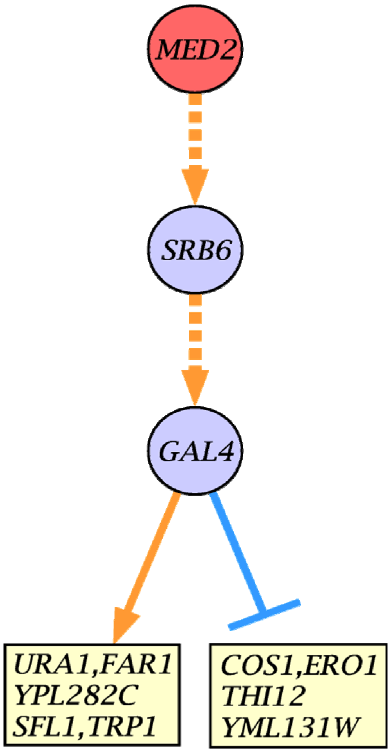

Model 31

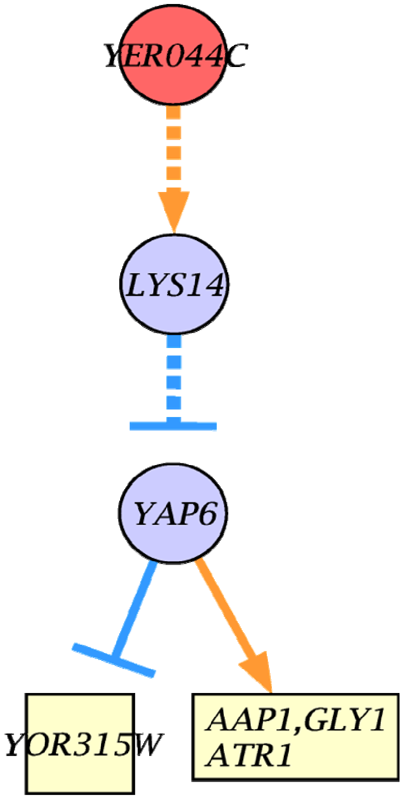

Model 32

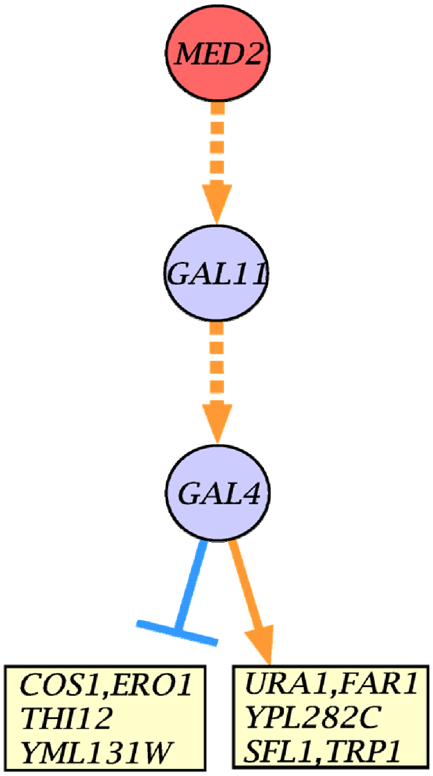

Model 33

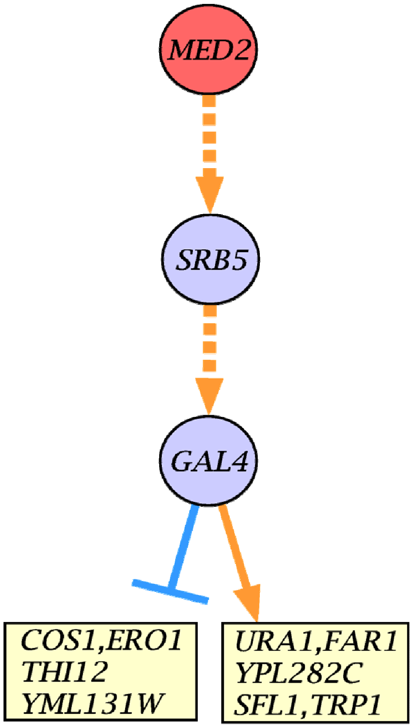

Model 34

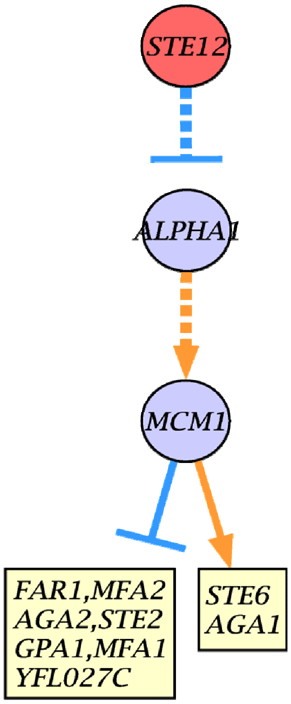

Model 35

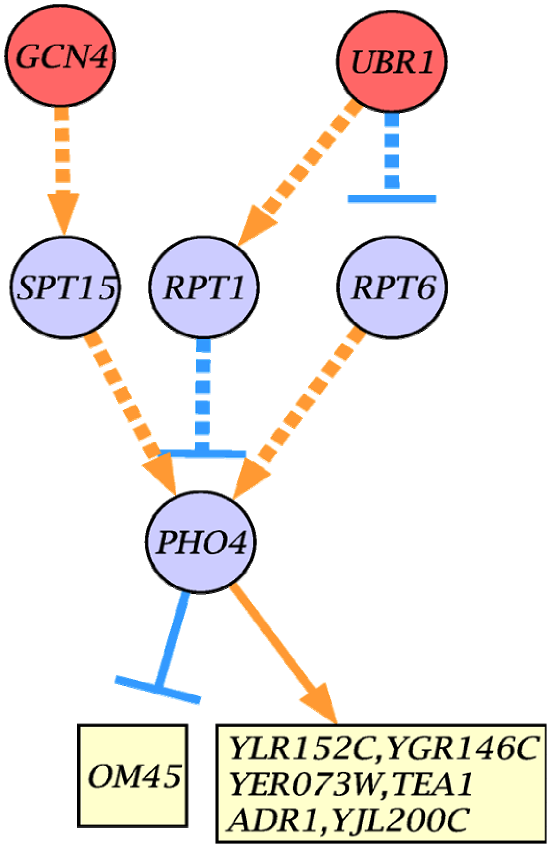

Model 36

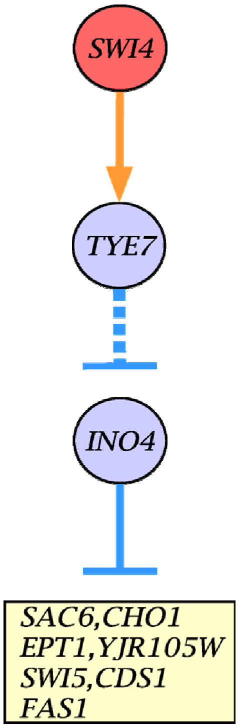

Model 39

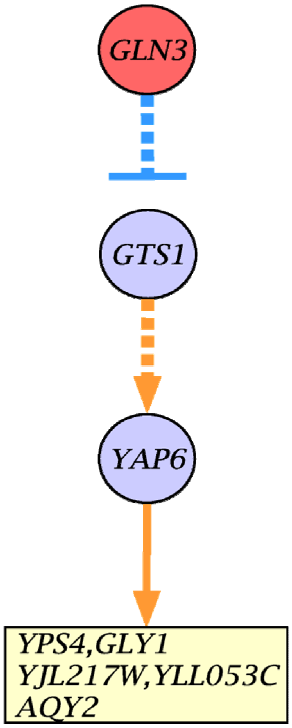

Model 40

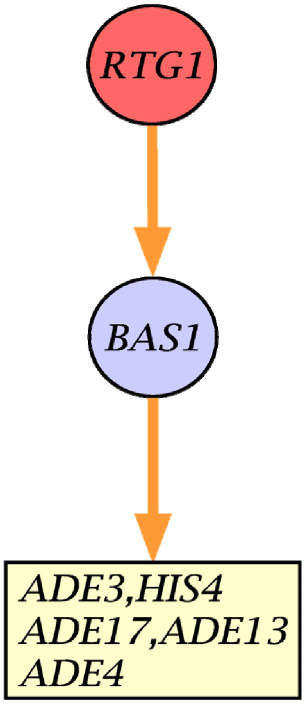

Model 41

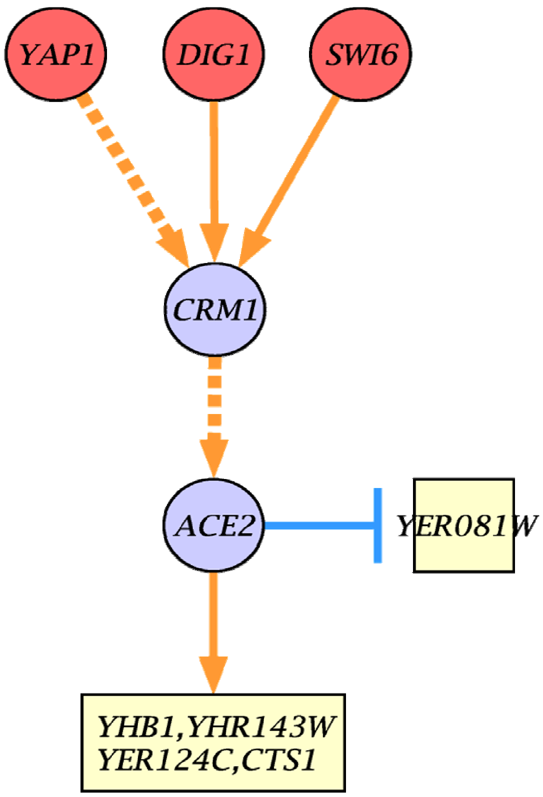

Model 42

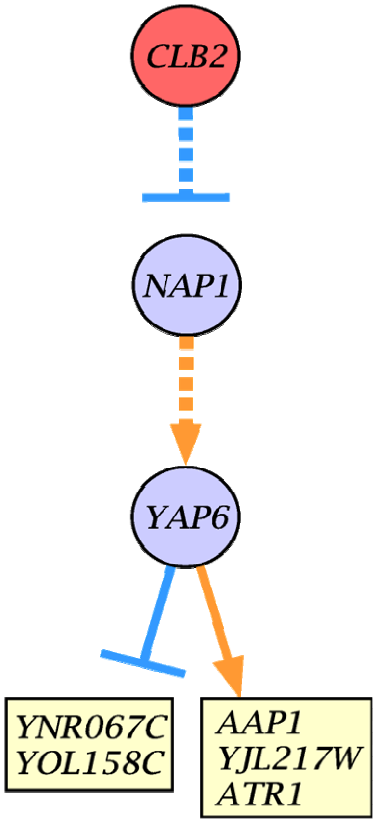

Model 44

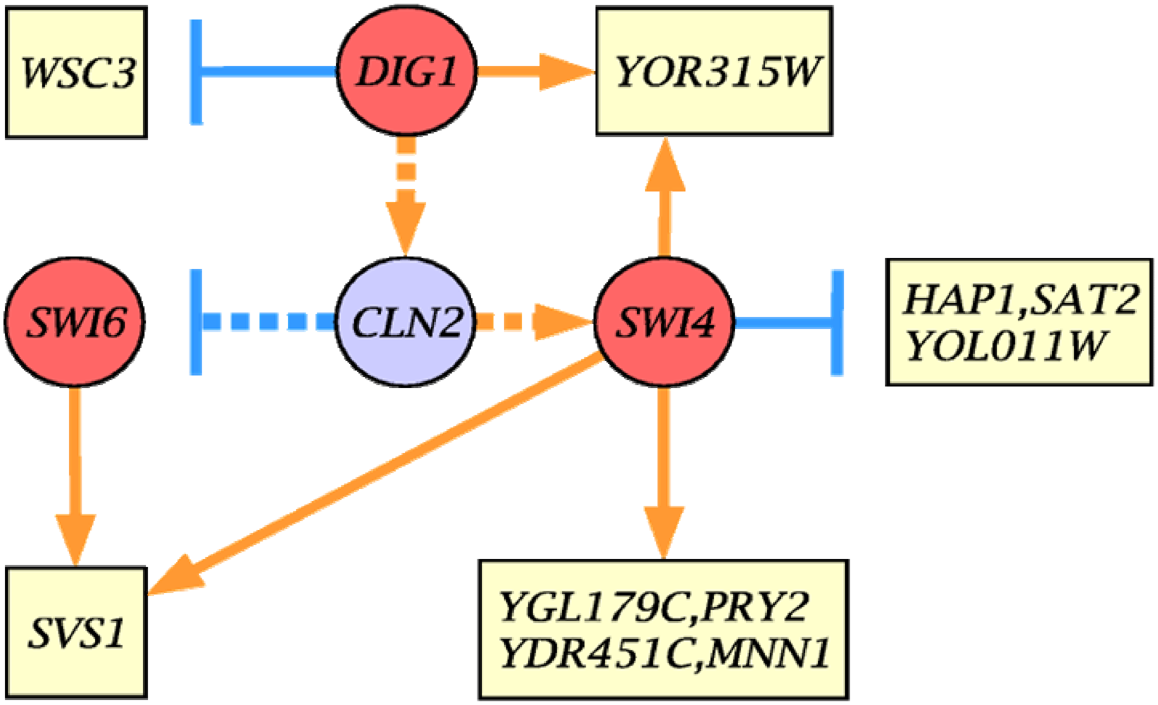

Supplement: Additional File 1 — Table S1 gives the internal validation for 17 out of 24 restricted network models; Table S2 lists the correlations between swi4δ and gcn4δ data and Rosetta and the new experiments; Table S3 gives the restricted subsets used to evaluate the reproducibility; and Table S4 gives the gene sets for external validation. [file gb-2005-6-7-r62-S1.pdf]
